# Deathly Accidents While High-Altitude Mountaineering in the Swiss Alps—An Observational Analysis from 2009 to 2021

**DOI:** 10.3390/ijerph191912498

**Published:** 2022-09-30

**Authors:** Benedikt Gasser

**Affiliations:** Departement für Sport, Bewegung und Gesundheit-Abteilung Rehabilitative und Regenerative Sportmedizin-Universität Basel-Grosse Allee 6, CH-4052 Basel, Switzerland; benedikt.gasser@yahoo.com

**Keywords:** sudden cardiac death, acute mountain sickness

## Abstract

Background: High-altitude mountaineering has become more and more popular. While many enjoy the beauty of the highest parts of Switzerland, there are considerable risks, which can even result in death. This study analyzed fatal events while high-altitude mountaineering in the Swiss Alps. Materials and Methods: In this study, cases of emergencies while high-altitude mountaineering in the Swiss Alps were analyzed in the period from 2009 to 2021 from the Swiss Alpine Club (SAC) emergency registry. Fatal emergencies were identified and analyzed in detail. Results: In total, 5020 emergency cases were analyzed, and among them 303 deathly events where detected. Of the fatal emergencies, 261 cases (86.1%) were male and 42 (13.9%) were female. The average age was 53.2 ± 19.1 years. More than half of the emergencies were on a route to a classic four-thousander. Fatal events were most common on the Matterhorn, with 40 cases (13.2%); on the Mönch, with 18 cases (5.9%); and on the Piz Bernina, with 10 cases (3.3%). In 245 of the fatal emergencies (80.9%), a fall was the cause. The second most prominent cause was rockfalls, with 16 cases (5.3%), followed by stranding, with 10 cases (3.3%), and avalanches, with 9 cases (3%). Illnesses and crevasse accidents counted together for less than 5% of the fatal cases. Almost two-thirds of fatal falls occurred while descending. Concerning nationality, 30% were from Switzerland and more than three-fourths of victims were from the countries of the Alps. Discussion: We found that falls were the most common cause of fatal emergencies in the Swiss Alps. Concerning the fact that most of these emergencies occurred during descents, fatigue and inadequate focus (forgetting the risks of the descent after successfully reaching the peak) are potential reasons for the fatal events. This potentially resulted from a lack of acclimatization, insufficient physical fitness, and inadequate tour planning. Since most victims were from the countries of the Alps, training tours may be possible as a recommended preparation for more difficult four-thousander peaks.

## 1. Introduction

High-altitude mountaineering has become more and more popular [1,2,3]. Over 40 million hikers and skiers visit the mountainous regions of the Alps per year, and it has been estimated that there are roughly 100,000–200,000 alpinists active per year in the highest parts of Switzerland [4]. High-altitude mountaineering is an excellent stimulation of the cardiopulmonary and skeletal muscle system over several hours [5,6,7]. Furthermore, relaxing in nature, being together with friends, or putting up a cross on a peak are motivating activities in engaging in a healthy lifestyle. The physical demands are high [8,9,10] and mountaineering has optimal effects on the cardiac system and aerobic metabolism [11,12]. However, these fundamentally positive effects on health, general wellbeing, or sense of life stand in contrast with the risks of the mountains and the non-negligible residual risks associated with high-altitude mountaineering. It is generally accepted and supported by analyses by the SAC (Swiss alpine club) and others that those who mountaineer above 4000 m are exposed to higher risks than mountain hikers or backcountry skiers [1,13,14]. Falls have especially attracted attention as a potential cause of fatal events, as prominent examples have existed since the beginning of alpinism, such as the tragedy of the first Matterhorn ascent. However, it is tempting to assume that today, with the development of well-secured routes, there are more common causes of emergencies and fatal events while high-altitude mountaineering beside falls [1,13,15]. In addition, the enormous development of tools for tour planning, improved weather forecasts, and widely available route information on the Internet should make emergencies less likely and less severe. Furthermore, as a consequence of tools such as the new REGA app (Swiss Air rescue organization), emergency services can locate alpinists (in most terrains) directly from helicopter [16,17,18]. As a consequence, fatal events should have become less likely and the risk perception of mountaineering may also have been lowered. To sum up, technological might yield to a tendency of a decrease in fatalities. This is supported by the findings of a former analysis from rock climbing of the mountain rescue services in Germany, Austria, southern Tirol, Zermatt/Switzerland, and Chamonix/France from 1987 until 1997, indicating that the number of fatalities retrieved during such rescue missions showed no significant increase [19]. Furthermore, older analyses of the SAC support the findings of this tendency of a decrease in fatalities [1,13]. This is somewhat intuitive, as the possibilities for emergency actions, such as faster alarming, faster localizing, and faster emergency actions, have improved. Furthermore, weather forecasts have improved, routes are better secured, and general material has improved. All of these considerations lead to the aim of this study to systematically analyze the development of fatal mountain accidents in the Swiss Alps over recent years concerning their number, causes, and mechanisms. We hypothesize that the number of cases did not change over the observational period [20].

## 2. Methods

### 2.1. Analyzed Population

All cases of high-altitude-mountaineering emergencies in the SAC central registry from the period of 2009–2021 were considered in this analysis. The central registry contains data from the Swiss Air Rescue Service (REGA), Air Glaciers Lauterbrunnen, Air Glaciers Saanenland, the Register SAC, the KWRO (Kantonale Walliser Rettungsorganisation), the Snow and Avalanche Research Institute Davos, and the cantonal police registers. The term mountain emergency covers any event where mountaineers ask for help from mountain rescue services or are affected by mountain hazards [21,22,23]. Each mountain emergency in the registry included an emergency number, the date, rescue organization, event, place, canton, activity, a NACA score (National Advisory Committee for Aeronautics score), the nationality of the victim, their birth date, sex, place of residence, the coordinates, and a short report [24,25]. We analyzed all fatal cases in detail. Ethical approval for secondary data analysis was obtained from the Ethics Committee of Northwestern and Central Switzerland (EKNZ).

### 2.2. Data Preparation

The causes of mountain emergencies were classified. In the 14-year period from 1 January 2009 (start of the registry) to 31 December 2021, the central registry encompassed a total of 5020 cases where mountain rescue services responded to high-altitude mountaineers in the Swiss Alps. Fatal cases were analyzed in detail with regard to age, sex, nationality, the time of occurrence, causes, and mechanisms.

### 2.3. Statistical Analyses

Mean and standard deviation of the age for the entire sample and separated for male and female subjects were calculated. To analyze potential differences in age in male versus female subjects, Jarque–Bera tests [26] were performed to determine if there was a normal distribution. Mann–Whitney U tests had to be performed to detect potential differences, as there was not a normal distribution. To analyze changes in the number of cases over the observation period, linear regressions with calculations of the coefficient of determination (*R*^2^) were performed for the whole sample, for female and male subjects, and for the main subclasses (falls, rockfalls, being stranded, avalanches). Calculations were made with Microsoft Excel (Microsoft Inc., Redmond, WA, USA) and SPSS 22 (Armonk, New York, NY, USA).

## 3. Results

**Figure 1:**
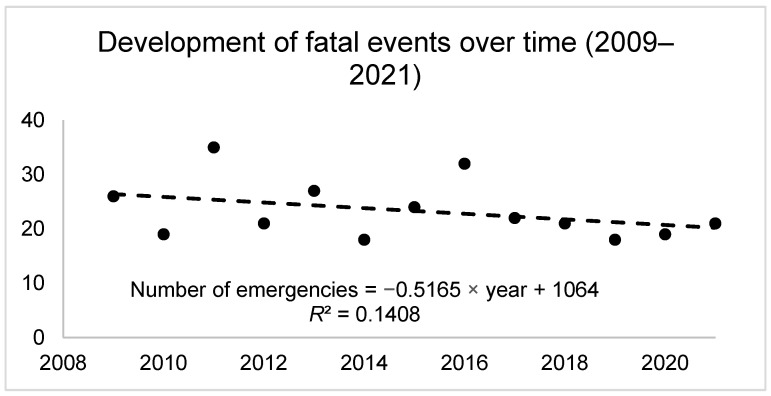
Fatal emergencies over the observational period (2009–2021). The suspected number of fatal events declined even more in relative terms. If one takes the SAC estimates of 150,000 alpinists in 2013 and of an increase of 4% per year, then there are around 225,000 alpinists per year now.

In the analyzed sample of 5020 emergencies, a total of 303 deathly events (6.03%) were detected. Of those cases, 261 (86.1%) were male and 42 (13.9%) were female. The average age of the total sample was 53.2 ± 19.1 years. The average age was 49.4 ± 14.3 years in females and 53.3 ± 18.9 years for males, so there was no significant difference between the sexes (*p* = 0.347). For around 80% of the fatal emergencies, a fall was the cause, with 18.8 cases per year (see Table 1). The second most prominent reason was rockfalls, followed by becoming stranded and avalanches. Illnesses and crevasse accidents counted for less than 5% of fatal cases. A decline in the number of fatal cases was detected over the observational period (see Figure 1). Interestingly, the share of fatal cases in relation to total emergency cases also decreased (see Supplemental Figure S1). However, while fatal emergencies from falls, rockfalls, and avalanches declined, the number of fatal emergencies due to becoming stranded increased (see Supplemental Table S1). The pattern of a decline is further seen for both sexes (see Supplemental Table S2).

**Figure 2:**
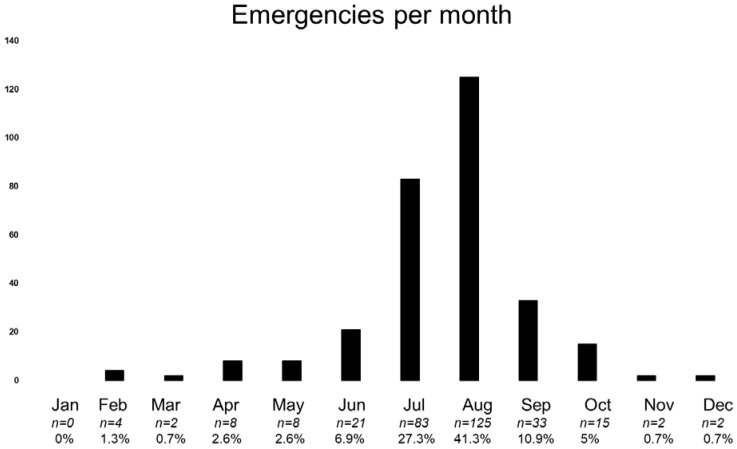
Most fatal events were in the summer months of July and August.

A total of 172 cases were detected (56.8%) on classic four-thousanders. The most common peaks were the Matterhorn with 40 fatal cases (13.2%), the Mönch with 18 (5.9%), and the Piz Bernina with 10 (3.3%). Concerning region, the cantons with the most cases were Valais (203 cases, 67%), Bern (49 cases, 16.2%), and Grisons (36 cases, 11.9%). A significant number of other cases were in the small mountainous cantons of Uri (4 cases, 1.3%), Glarus (3 cases, 1%), Ticino (2 cases, 0.6%), and Obwalden (1 case, 0.3%), so in total around 95% of the cases were in a mountainous canton. The rest were in border terrain. More than two-thirds of the cases were in the summer months of July and August (see Figure 2).

Of the 245 fatal emergency cases due to falls, detailed analyses of case reports revealed that 72 cases concerned a team of two alpinists in which both were victims, so these cases were actually due to 36 separate events. Another 18 fatal cases were due to falls where teams of three alpinists were all victims (six events), and one fatal fall event was the cause of death for a team of five alpinists. In these 95 cases (38.8% of fatal emergencies due to falls), the entire team was likely dragged down when one of its members fell. In another 110 cases (44.9%), the alpinists were either alone or were in a group but not roped. In 18 of the fatal emergency cases from falls, there were clear indications in the case reports that the use of a rope might have prevented the fatal event. In 35 of the fatal cases from falls (14.3%), the mechanism is not known, and in some of these cases, very limited speculation is possible because the victims have not been found, though sometimes some equipment, such as ice axe, has been recovered. Nevertheless, together, the case reports indicate a typical pattern: after a successful and sometimes very challenging tour, alpinists descend without ropes and fall. For example, in one case, an alpinist fell while going down the Hörnligrat unroped after completing the north face of the Matterhorn. Of all fatal falls, 61.1% were during descents as in this example, and only 38.8% were during ascents.

Concerning the 16 fatal emergency cases due to rockfalls (5.3%), a team of two alpinists were both hit by a rockfall in three separate events, a large scree avalanche was the cause of another two fatalities, and a large rockfall fatally injured a team of three, two of whom were hit on the head. Concerning the 10 fatal cases of being stranded (3.3%), fog and weather changes were the main causes mentioned in the case reports. Of the nine fatal cases due to avalanches (3%), six were because of slab avalanches and two were because of wet avalanches. Six fatal emergency cases were due to an illness (2%), three were potentially attributable to AMS (diagnosed with Lake Louise Score), and two were possibly attributable to sudden cardiac death [27]. Both of those victims were over 60 years old, and the average altitude was around 3500 m. Of the four fatal emergency cases due to a crevasse accident (1.3%), one is especially worth mentioning. On a glacier, two roped alpinists died while simultaneously crossing a snow bridge that collapsed. Three cases were due to material failure (1%), two to lighting strikes (0.7%), and one to being crushed (0.3%).

In 91 of the fatal emergency cases (30%), the victims were from Switzerland; in 82, the victims were from Germany (27.1%); in 35, the victims were from Italy (11.6%); in 17, they were from France (5.6%); and in 9, they were from Austria (3%). More than three-fourths of the victims were from the countries of the Alps. Of the other victims, 11 were from the Czech Republic (3.6%), 10 were from Poland (3.3%), 8 were from Great Britain (2.6%), 7 were from Japan (2.3%), 6 were from Belgium (2%), 5 were from the Netherlands (1.7%), and 4 were from Estonia (1.3%). Countries with less than three fatal emergency cases (<1%) were the United States, Romania, Slovakia, Denmark, Croatia, Latavia, Chile, and Slovenia.

## 4. Discussion

The aim of this study was to give an overview of fatal emergencies in the Swiss Alps in the period from 2009 to 2021. Of the total 5020 emergency cases analyzed, 303 (approximately 5%) were identified as fatal, which is in line with findings from the Austrian alps, indicating a case fatality rate of 4.7% while rock climbing [14]. On average, there were 23 fatal cases per year. Falls were the main cause, followed by rockfalls, being stranded, avalanches, illnesses, and crevasse accidents. Very rare events such as material failure, lightning strikes, or being crushed counted for less than three cases each in the total period. These findings are in line with those from an analysis from the Austrian Alps elucidating injuries in rock climbing, whereby falls were similar to the findings here the main cause, followed by rockfalls [14]. Embedding this findings broader, an around ten year-old review elucidated injuries in mountaineering, rock and ice climbing and detected in addition falls as the main reason for emergencies [28]. Here, a decline in the number of cases was detected for the main classes of falls, rockfalls, and avalanches, but not for being stranded, which is in line with findings from other countries and which allows us to reject the initial hypothesis that the number of events did not change over time [14,15,29]. Summarizing the results and those of others, the trend of a decline of fatalities seems to have continued since the mid-eighties until today [1,13,14,19]. The average victim was around 50 years old and male, which might be, besides other reasons, due to the fact that the physical peak was reached and a decline in physical capabilities can be presumed. Most fatal accidents happened during the summer season; in most cases of fatal falls, there was a team of two alpinists, and fatal accidents were more likely during descents than during ascents [21,22,30]. Since almost 90% of the victims were male, it seems likely that men have a lower risk aversion in the mountains and that this predisposes them to fatal events. As there were a significant number of fatal emergencies where a rope team was dragged down, the pure difficulty of a tour does not seem to be the most relevant factor for predisposing one to a fatal event. This is supported, for example, by the finding that fatal events were identified on a number of relatively easy tours, such as on the route to the Mönch from the Mönchsjochhütte, which points to another risk factor: insufficient acclimatization. Since most cases in which the emergency mechanism was clear were reported on a descent, insufficient acclimatization resulting in fatigue and stumbling is a likely mechanism. For example, a stumble when a crampon is caught on some clothing or on the rope can lead to a fall, and if the rope team cannot hold on, they are dragged down as well. During descents, alpinists are often not only fatigued, they are also sometimes going down face forward. As a consequence, a fall or stumble is directed away from the mountainside, which quickly accelerates the fall. By contrast, in ascending or descending backward with one’s face toward the mountainside, one usually falls on one’s hands, which increases the chances of catching one’s fall at the beginning.

As a practical implication, it is important to mention that since many fatal falls occurred during descents, there seems to be a high preventive potential, especially when they occurred in mixed terrain with stretches of climbing rated II–III UIAA (Union Internationale des Associations d’Alpinisme) and when a rope was not used. For example, there were a number of cases on the Hörnligrat, where the typical climbing sections have a difficulty of around III. Going on rope on these sections and using a short-rope technique can prevent stumbles from becoming fatal falls [21,22]. For example, one can anchor around the rock and the person belaying can hold the rope firmly to immediately stop a stumble from developing into a fall. More than half of the fatal emergencies were on routes to a classic four-thousander, and all these mountains have stretches where walking on a short rope is necessary [21,22]. Furthermore, the fact that cases were more likely on descents implies that fatigue played a role. Proper training and adequate acclimatization are helpful in preventing fatalities [4,23,29].

In addition, rockfalls declined over time. Intuitively, one might think that these events would be becoming more common due to climate change and melting permafrost, but this is not supported by the numbers. The mechanism of some of these cases might have been a residual risk existing while high-altitude mountaineering. Fatal emergency cases of being stranded was the only class that showed an increase in the number of cases over the observational period. New media technologies, YouTube, and Twitter bring astounding images of tours into homes, but they only convey the dangers in a limited way, which could lead to people engaging in riskier behavior [17,21,22]. Avalanches can occur even in high-altitude terrain. In six cases, a slab avalanche was responsible for the accident, and in two cases a wet avalanche was responsible. It is tempting to assume that in these cases, risky behavior was partly responsible for the deathly event. Astonishingly, crevasse accidents are very seldom. Maybe this is due to the fact that it is more common to follow the principle of using a rope on a glacier, which results in fewer deathly emergencies.

When looking for limitations of the study the high degree of uncertainty in the case reports is to mention. Case reports were normally written shortly after an event by the emergency services and were drafted normally relatively short. Furthermore, substantial reasons can be mentioned for the limited accuracy of the findings. For example, we do not know if a person had a sudden cardiac death that caused a fall. For practical reasons, autopsies are normally not performed, so such a case would be counted as a fall even though the principal cause was cardiac death [31]. When trying to elucidate mechanisms, a prospective design would be desirable in order to improve the evidence, meaning that an in-depth analysis is conducted immediately after an emergency with, for example, the interviewing of alpinists that alarmed the emergency services.

## 5. Conclusions

To summarize, fatal accidents while high-altitude mountaineering have decreased in recent years. This stands in contrast to non-fatal events due to being stranded (main class of non-fatal events), which have increased in total numbers [15,29]. The decrease in deathly events is thus somewhat surprising, in particular when one considers the increasing number of alpinists active in the Swiss Alps. Still, when focusing on practical implications, while fatal events have become less likely, some evidence suggests that there is inadequate risk aversion: for example, there were more male than female victims; accidents primarily occurred while descending; often a whole team of alpinists died, so partners might not have been securing correctly; there were cases due to a wet avalanche; there was a high number of cases on famous mountains such as the Matterhorn or Piz Bernina; and alpinists sometimes did not tell relatives exactly what tour they were undertaking. Together, all of this suggests that there might have be a lack of carefulness and awareness of the risks of alpinism [21,22]. In practice, adequate acclimatization, proper advance training for the tour, and precise tour planning with clear statements about the route being made to relatives should be understood as basic ways to reduce the residual risks of high-altitude mountaineering. To learn these skills, undergoing courses offered, for example, by the SAC or the Alpine Rettung Schweiz is recommended.

## Figures and Tables

**Table 1:** Causes of deathly emergencies while high-altitude mountaineering in the Swiss Alps. Being stranded (or blocked) is a mountain emergency where alpinists can no longer continue their tour on their own due, for example, to exhaustion, equipment problems, or weather [16,17].

Cause	*n*	Percent	Average Cases per Year
Fall	245	80.9%	18.8
Rockfall	16	5.3%	1.2
Stranded	10	3.3%	0.8
Avalanche	9	3%	0.7
Illness	6	2%	0.5
Crevasse accident	4	1.3%	0.3
Material failure	3	1%	0.2
Lightning	2	0.7%	0.2
Crushed	1	0.3%	0.1
Other	7	2.3%	0.5
Total	303	100%	23.3

## Data Availability

Data is available on qualified request.

## Supplementary Materials

The following supporting information can be downloaded at: https://www.mdpi.com/article/10.3390/ijerph191912498/s1. Supplemental Table S1: Development of the number of cases for each subclass over the observational period calculated with linear regression models of the form number of cases = alpha + beta * year with calculating the coefficient of determination. Supplemental Table S2: Development of the number of cases for men and woman over the observational period calculated with linear regression models of the form number of cases = alpha + beta * year with calculating the coefficient of determination. Supplemental Figure S1: Development of the share of fatal events over the observational period.
