# Supplementary material for: Deathly Accidents While High-Altitude Mountaineering in the Swiss Alps—An Observational Analysis from 2009 to 2021
Source: Int J Environ Res Public Health. 2022 Sep 30;19(19):12498. doi: 10.3390/ijerph191912498 (PMC9566316; doi:10.3390/ijerph191912498)
Supplement: Supplementary file 1 [file ijerph-19-12498-s001.zip › ijerph-1922263-supplementary.pdf]

Supplemental material

Supplemental Table S1. Development of the number of cases for each subclass over the observational period calculated with linear regression models of the form number of cases = alpha + beta * year with calculating the coefficient of determination.

	**alpha**	**beta**	R^2
Stranded (*n* = 10)	-132.9	0.066	0.077
Avalanches (*n* = 9)	189.2	-0.093	0.050
Rockfalls (*n* = 16)	200.4	-0.099	0.066
Falls (*n* = 245)	1115	-0.544	0.163

Supplemental Table S2. Development of the number of cases for men and woman over the observational period calculated with linear regression models of the form number of cases = alpha + beta * year with calculating the coefficient of determination.

	**alpha**	**intercept**	R^2
Woman (*n* = 42)	-0.154	313.2	0.103
Men (*n* = 261)	-0.379	784	0.114

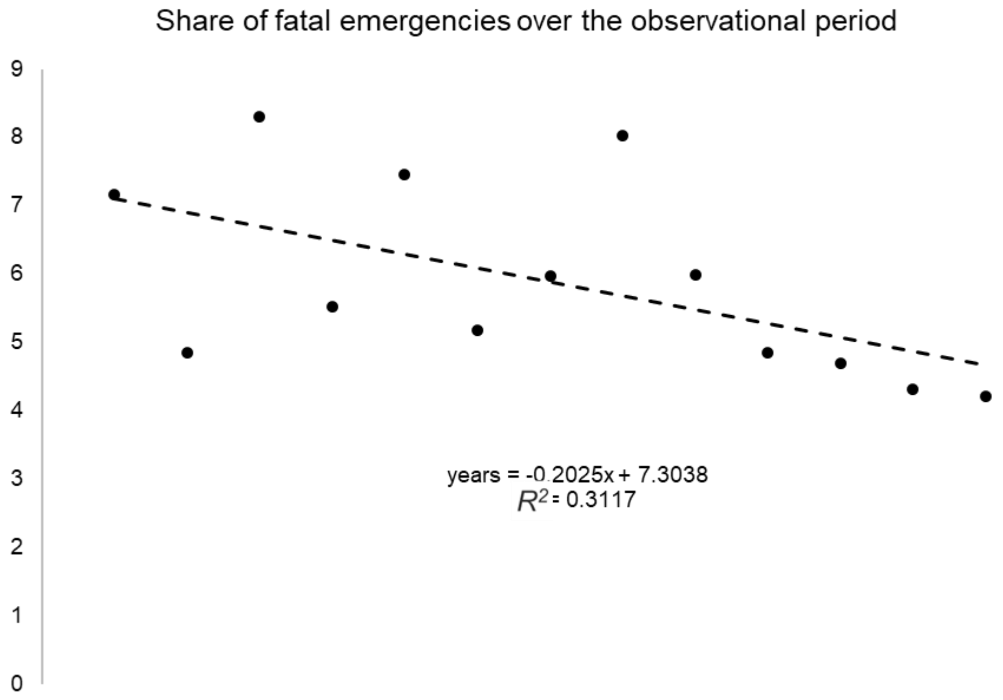

Supplemental Figure S1. Development of the share of fatal events over the observational period.
